# Evaluation of a Virtual Health Hub for People Experiencing Homelessness in Sydney, Australia: Ensuring Physical and Psychological Primary Health Care in Crisis Accommodation

**DOI:** 10.3390/ijerph21121593

**Published:** 2024-11-29

**Authors:** Cathy O’Callaghan, Paul Clenaghan, Alenda Dwiadila Matra Putra, Fiona Haigh, Sue Amanatidis, Freya Raffan, Nicole Lynch, Margo Barr

**Affiliations:** 1 International Centre for Future Health Systems (ICFHS), Faculty of Medicine and Health, University of New South Wales (UNSW), Sydney, NSW 2052, Australia; alenda_dwiadila_matra.putra@unsw.edu.au (A.D.M.P.); f.haigh@unsw.edu.au (F.H.); margo.barr@unsw.edu.au (M.B.); 2 Clinical Services Integration and Population Health, Sydney Local Health District (SLHD), Sydney, NSW 2050, Australia; paul.clenaghan@health.nsw.gov.au (P.C.); nicole.lynch@health.nsw.gov.au (N.L.); 3 RPA Virtual Hospital (Rpavirtual), Sydney Local Health District (SLHD), Sydney, NSW 2050, Australia; sue.amanatidis@health.nsw.gov.au (S.A.); freya.raffan@health.nsw.gov.au (F.R.)

**Keywords:** virtual health care, homelessness, health access, health equity, primary health

## Abstract

Introduction: Individuals experiencing homelessness have higher rates of chronic disease and face challenges accessing primary care. Virtual health care can reduce health inequity but needs user acceptance. A virtual health hub (VHH) for people experiencing homelessness in Sydney provided virtual GP and psychologist care within a crisis accommodation service. This included nursing assistance. Method: The VHH evaluation assessed the feasibility of the service specifically examining accessibility, efficiency, costs, technology, quality, and outcomes through attendance data, patient measures, stakeholder interviews, and case studies. Findings: Data indicated 40% client utilisation with high attendance for GPs and/or psychologists. All clients reported a high quality of care, appointment benefits, understanding clinicians, and treatment help, and that privacy was maintained. If the VHH was not available, one-third would not have sought treatment. The majority agreed that virtual care was the same or better than in-person care. Only a few experienced technical issues. Service provider interviews indicated the benefits of accessible and affordable care, perceived reduced hospital presentations, staff time saved, and reduced client costs. Limitations were the lack of physical examinations and lack of follow-up due to temporary accommodation. Strong stakeholder partnerships enabled implementation success. Conclusions: The VHH service is feasible and replicable with on-site assistance and stakeholder commitment.

## 1. Introduction

Homelessness in Australia is defined as “when a person does not have suitable accommodation alternatives” and if their current living arrangement is in a dwelling that is inadequate or has no tenure, or if their initial tenure is short and not extendable, or does not allow them to have control of, and access to, space for social relations [1]. This includes people living in boarding houses and crisis accommodation. In 2021 it was estimated that 122,494 people were experiencing homelessness in Australia (48 people per 10,000) [2]. Individuals experiencing homelessness face a range of complex health needs, and higher rates of chronic and mental health conditions, and mortality [3,4,5,6]. They experience barriers accessing and receiving necessary health services [5,7,8] due to affordability, availability, competing priorities, and stigma [9,10]. A lack of access may also mean higher use of acute services [11,12]. There are different models of care that use collaborative multi-agency approaches to provide accessible health care to people who are homeless, such as mobile clinics and outreach services to people residing on the street or in temporary crisis accommodation/shelters [13,14,15,16].

Virtual health care is a convenient way for clients to connect with health professionals using video or phone technology [17] and has become a more viable option for communities since the COVID-19 pandemic [18]. Virtual health care has the potential to reduce health equity concerns, addresses access and availability of services for different populations [19,20,21], including homeless populations with complex health conditions, which requires multifaceted solutions to address issues of digital literacy, access to technology, on-site assistance and private spaces [22,23,24]. However, there are concerns that mainstream virtual care services could exacerbate existing health inequities if these services are not developed to be accessible, available, and acceptable to vulnerable populations [21]. Studies assessing the use of technology in health care provided to populations experiencing homelessness show clients are enthusiastic about such models, but providers are more hesitant about their feasibility [25]. The use of free mobile phone technology has also raised issues of client trust in providers and concerns regarding the use of their private information [26]. Other research on virtual care discusses the importance of rapport between provider and client and suggests that clients may perceive relationships and rapport as more important to treatment experience than modality [27].

A pilot project of a virtual health hub (VHH) for people experiencing homelessness in a health district in metropolitan Sydney was developed to provide place-based virtual care within inner-city crisis accommodation. This region holds a substantial population of individuals experiencing homelessness and residing in boarding houses [15]. Although on-site general practitioner (GP) services in homeless environments are crucial, they often operate at capacity, and GPs experience barriers accessing medical specialists for this population [28]. The VHH program acknowledged the challenges faced by people experiencing homelessness in accessing and receiving necessary medical services, including virtual healthcare [22]. The VHH Model of Care trial was a collaboration between the Homeless and Rough Sleepers Program within Clinical Services Integration and Population Health and RPA Virtual Hospital (rpavirtual) in Sydney Local Health District (SLHD) and the crisis accommodation setting. The project was funded by SLHD with the view of expanding to other sites frequented by this population. Virtual care was only one aspect of the services available to clients at the accommodation that were evaluated in the project.

The VHH Model of Care trial aimed to improve access to health care for people who experience homelessness when access to traditional primary care is limited, enhance engagement and collaborative care with partners who work with people who experience homelessness, develop a multi-agency framework for homelessness service to deliver health care in a culturally safe manner for people who experience homelessness, and commit to the provision of virtual health care and the required information and communications technology (ICT) infrastructure [28]. 

Initially, the virtual services offered by the program were designed to complement the existing on-site medical services, creating a comprehensive healthcare solution for populations at risk of or experiencing homelessness. In this model, the on-site GP would be the lead primary health doctor, while the VHH GP would provide complementary support (e.g., when the on-site GP was unavailable or at capacity). However, the on-site GP resigned from the crisis accommodation setting prior to the beginning of the VHH implementation. The crisis accommodation was unable to recruit an on-site GP, so the VHH model evolved to become the only way for clients to access primary care. The evaluation study examined whether the VHH worked as intended and how it addressed gaps in health care for populations experiencing homelessness.

As part of the evaluation, the domains of accessibility, cost, quality of care, technological infrastructure, client and stakeholder experiences, and outcomes and impact were assessed to analyse the extent to which the VHH effectively reached and served the target population [29]. As the intervention was a pilot and the evaluation was exploratory in nature, short-term implementation, service and client outcomes were considered rather than longer-term client outcomes.

The evaluation project was overseen by an evaluation working group (WG), whose role was to recommend an evaluation framework and provide advice on the project design, data collection, and analysis to the overall project’s implementation task force and management committee. The group consisted of representatives from UNSW, the Homeless Program, and rpavirtual in SLHD, the crisis accommodation setting staff and a lived experience consultant. The ICFHS/CPHCE at the University of New South Wales (UNSW) led the evaluation of the VHH over 12 months, which was funded through SLHD. 

## 2. Materials and Methods

In the VHH program evaluation, we used mixed methods, including an evaluation framework and collection and analysis of quantitative and qualitative data. The evaluation framework consisted of a literature scan and program logic and was designed with the assistance of the evaluation WG. The evaluation framework informed the project design, methodology, and evaluation questions.

- Accessibility: To what extent can the VHH be accessed by clients residing at the crisis accommodation according to perceived affordability, acceptability, availability, and convenience?
- Quality of care: What is the standard of service delivered through the virtual health platform, including stakeholder and partner experiences, range of services provided, and clinical procedures?
- Technological infrastructure: What is the technological setup, connectivity, and reliability of the virtual platform, ensuring its suitability for delivering seamless virtual health services and the ability of all service users to use the system?
- Stakeholder experiences: What is the level of engagement and collaboration among service providers, including healthcare providers, partner organisations, and the target population, to identify opportunities for improvement?
- Cost: How efficient is the VHH model with resource utilisation, appointment management, and other aspects that may inform the cost of the VHH solution?
- Outcomes and impact: What are the short-term outcomes and impact of the VHH model in terms of overall client satisfaction?

**Figure 1:**
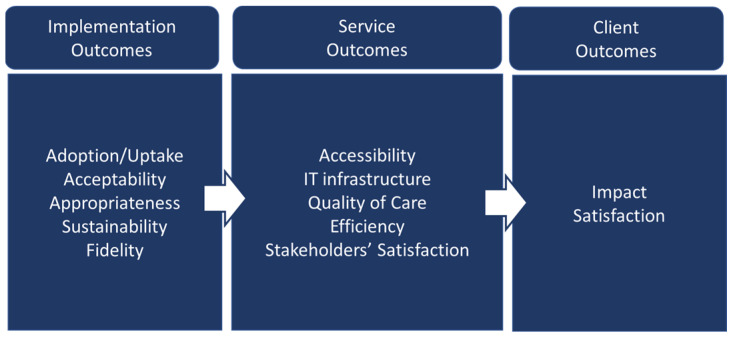
Types of outcomes used in VHH evaluation modified from Proctor’s et al. 2011 [29].

The evaluation WG adapted Proctor’s implementation outcome framework [29] into the VHH project to understand the implementation of the service (see Figure 1). This included the factors of adoption or uptake, acceptability, appropriateness, sustainability, and fidelity (how closely the implementation aligns with the initial plan). The service outcomes included accessibility, quality of care, technological infrastructure, stakeholder satisfaction, and cost. Lastly, the client outcomes covered impact and client’s satisfaction with the service. As this VHH service was a pilot project, it was essential to assess the implementation outcomes, not only the client or service outcomes. In implementation science and research, implementation outcomes serve as the preconditions for attaining the desired service delivery and clinical outcomes [29]. In this sense, the VHH team should successfully implement VHH as indicated by its acceptability, appropriateness, sustainability, adoption, and fidelity to achieve good service and client outcomes.

Quantitative data included VHH appointment records and Patient Reported Experience Measure (PREM) survey responses. This information provided insights on the adoption and sustainability of VHH implementation in the crisis accommodation as well as quantitative descriptions of clients’ satisfaction with the VHH service. Qualitative data collection and analysis included service providers’ perspectives and experiences in working together to implement the VHH service, as well as perceived service and client outcomes through in-depth interviews. The details of each data collection and analysis method are explained further in Section 2.2 and Section 2.3. The relationships between data sources and outcomes used in this study can be seen in Table 1.

### 2.1. Setting

The VHH was delivered in the Haymarket Centre, located in SLHD, New South Wales (NSW), Australia. The Haymarket Centre is a 24-bed crisis accommodation (average length of stay is up to 3 months) and support service for people over the age of 18 years who are experiencing homelessness while living with co-occurring mental health issues, alcohol and other drug dependencies [30]. To be admitted into this service, clients must be homeless. Long-term housing and health solutions are found for people who are frequently homeless, and many of the residents have been turned away by other services. People experiencing homelessness can reside temporarily with or without a referral from external parties. This centre also provides services to establish and support safe housing pathways for residents with a goal of long-term and independent living.

The VHH model was led by the implementation team, which provided service delivery and had direct interaction with the clients in the crisis accommodation setting, consisting of a project nurse from the SLHD Homeless Team, case managers from the crisis accommodation setting, and health professionals from rpavirtual (see Table 2).

**Figure 2:**
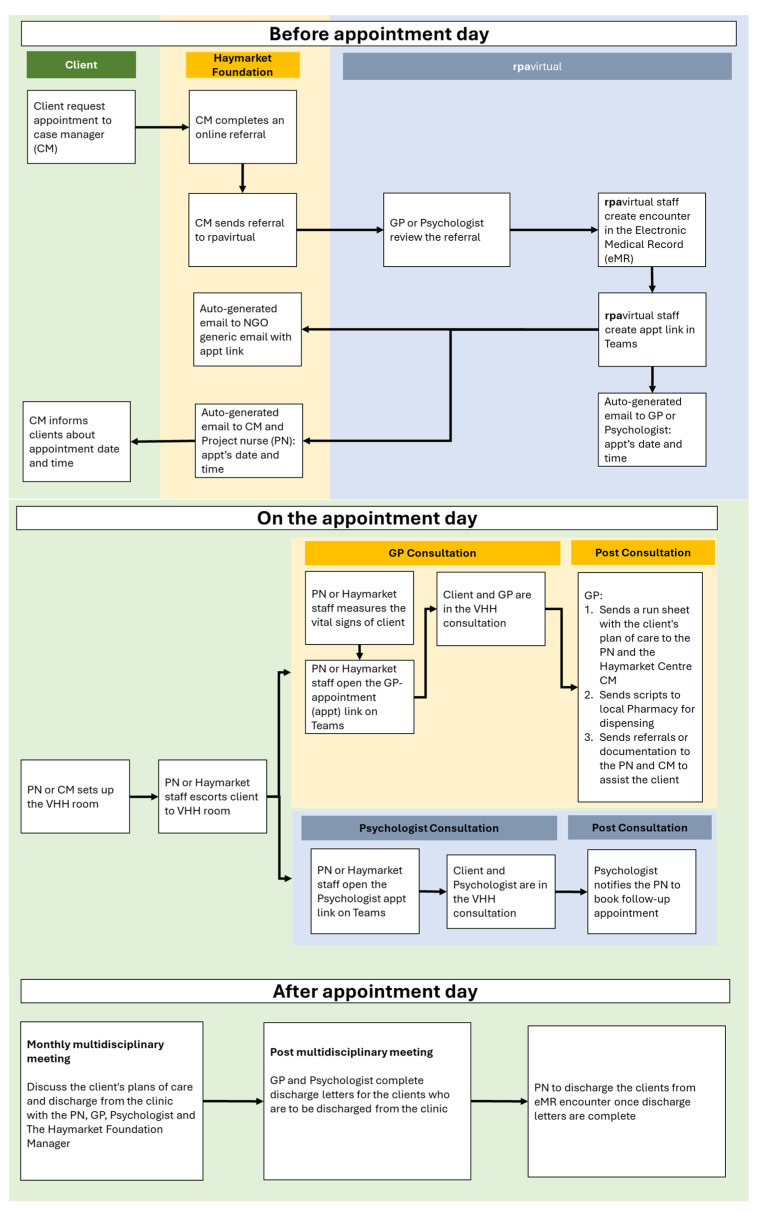
Client flow in VHH service.

The process for clients to make and attend a VHH appointment is indicated in the client flow diagram below (Figure 2).

### 2.2. Quantitative Data Collection and Analysis

The quantitative data sources included VHH appointment records and PREMs data of clients who accessed the VHH. This data was assessed using a descriptive approach which was generated through an appointment dashboard system. Between 1 August and 13 February 2024, there were 82 clients, consisting of 43 men and 39 women, who resided in the crisis accommodation setting. Of these 82 clients, 33 clients utilised the VHH.

People who experienced homelessness residing at the Haymarket Centre who used the VHH over the trial period were invited to complete a PREM survey as part of their usual care. The PREM was adapted from rpavirtual’s baseline patient experience survey and included validated questions from the NSW Bureau of Health Information. It consisted of 25 questions, including demographic, general patient experience, and virtual health experience measures. Clients completed the PREMs online using the online software Research Electronic Data Capture (REDCap) or via a paper copy with data entered into REDCap by the VHH clinicians. From 10 October 2023 to 29 February 2024, a total of 20 PREM surveys were completed from 33 clients accessing the VHH who had seen either a GP or a psychologist via rpavirtual, with a response rate of 61% (out of 33 clients). The PREM data was extracted and used to assess the acceptability, quality of care, and impact of the VHH model of care using R-studio to obtain a descriptive summary of the PREMs.responses. 

To assess how similar the experience of clients using the VHH in the crisis accommodation was to that of general populations using other virtual health services, the team conducted a statistical analysis of the data using Chi-square. A comparison was made between the VHH people experiencing homelessness PREMs and all PREMs collected from rpavirtual Virtual Care Centre models of care in the same timeframe, such as Acute Respiratory, Virtual Fracture Clinic, Virtual Rehabilitation, Psychology, Virtual Trauma, rpavirtual Emergency Department, Tuberculosis and the Wound Care Command Centre. Each PREM differed slightly in reflecting the specific model of care, so the comparison was limited to the core questions consistent across all rpavirtual PREMs. Ethics clearance was provided to use all this data to make the comparison. The results of nine PREM questions from the VHH group and comparison group were analysed in R-studio to obtain the degree of difference.

### 2.3. Qualitative Data Collection and Analysis

Qualitative methods included in-depth interviews with service providers to understand the participants’ constructed meanings and experiences in a holistic way [31]. Interviews elicit an in-depth understanding of the objectives of the study [32], capture context-specific knowledge, and allow people to answer questions accurately, freely, and openly [33]. A purposive sample of participants was identified by the evaluation working group based on their knowledge and experience in relation to the VHH. Twelve interviews were conducted with management (two managers of rpavirtual and two managers from the crisis accommodation setting), case managers (two at the crisis accommodation setting), healthcare providers (two psychologists, one general practitioner and one nurse), and one person with lived experience of homelessness. Questions for the interviews were developed in consideration of the research questions, including accessibility, quality of care, technological infrastructure, cost, and stakeholder experiences. The interviews were conducted via video conferencing and transcribed verbatim.

Attendance data was also used to compile case study vignettes to exemplify the nature and frequency of the treatment given to the clients in the VHH. The case studies are indicative of the type of care and the characteristics of the clients, rather than based on actual individuals to retain anonymity [34,35].

A thematic analysis was conducted to identify common themes [36] with the assistance of the qualitative software, NVivo 12 [37]. An interpretive phenomenological approach (IPA) was taken [38,39] to holistically investigate the service providers understanding and constructed meanings of experience [35], as well as acknowledge the experience and contextual knowledge of the researchers in the analysis process [40,41]. The transcripts of the data were coded into categories according to patterns in the research using a combined inductive/deductive analytic approach. The data was coded deductively according to study aims, research questions, and implementation framework, while inductive analysis was carried out by identifying codes, categories, patterns, and themes as they emerged. The research team met to review and assess the coding framework to ensure the validity of the findings. Any differences in the themes identified were discussed, and the themes were refined accordingly.

The evaluation project was approved by RPA Human Research Ethics Committee in SLHD (X23-0381 & 2023/STE03933). A separate ethics application was also submitted by rpavirtual to access the PREMs data.

## 3. Results

The VHH was evaluated to assess implementation outcomes, service outcomes, and client outcomes. These outcomes were evaluated using the VHH appointment records, PREM data, case studies, and service provider interviews.

**PREM respondents’ profile**

As presented in Table 3, the age of the clients who completed the PREM ranged from 18 to 64 years, with the majority aged 35 to 44 years old (50%); 45% self-reported as male, 55% as female, and none self-identified as non-binary. Eighty-nine percent of PREM respondents spoke English at home, and two respondents spoke languages other than English at home. Nil required an interpreter for their care.

### 3.1. Implementation Outcomes

The implementation outcomes of adoption, acceptability and appropriateness, sustainability, and fidelity were evaluated using PREM data and stakeholder interviews.

#### 3.1.1. Adoption or Uptake

Data from the VHH appointment records at the crisis accommodation setting between 1 August 2023 and 13 February 2024 indicated that 40% of clients utilised the VHH (*N* = 33). Of these 33 clients, 27 clients saw a rpavirtual GP Visiting Medical Officer (VMO), 17 clients saw a psychologist, and 18 saw both. In total, 33 residents booked 155 appointments, consisting of 106 GP and 49 Psychology appointments. The appointment attendance rate was very high.

The SLHD Homeless Team nurse played the main role in organising and setting up the VHH appointments and working closely with case managers.

*I don’t know if it would work without (the project nurse), because she’s the front facing person. She’s the person we talk to about all the appointments. … she’s been on site—she has given us a lot of advice from a nurse standpoint. She’s invaluable. I think we couldn’t lose her and also she’s very good with setting everything up … the technical issues might be a bit harder to manage without her.*

(Case manager)

*The biggest thing is, if [the clients] are going to virtual health, and it’s a different doctor every time they see them, but [at least] it’s the same nurse.*

(Lived experience client)

**Figure 3:**
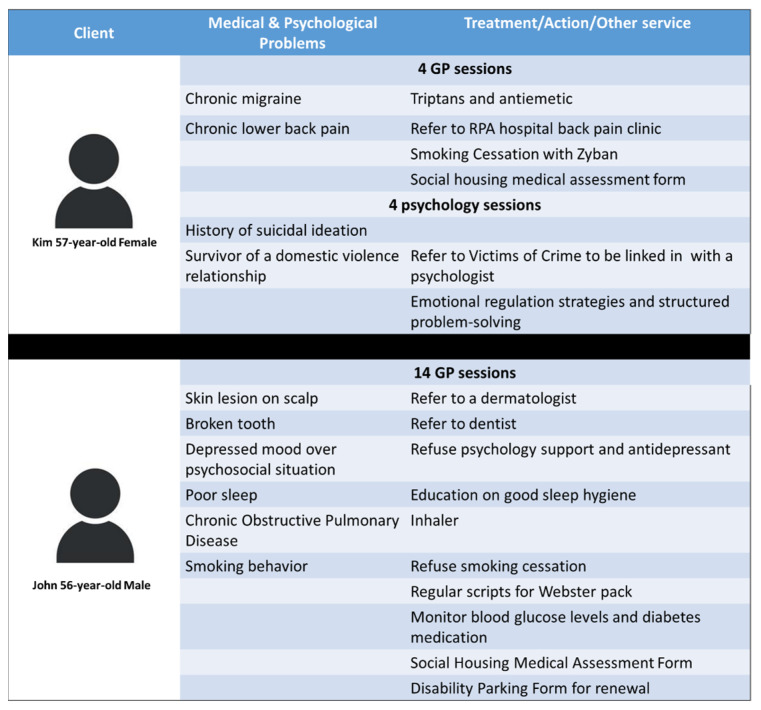
Two case study vignettes from the VHH.

The full adoption of the VHH by clients at the Haymarket Centre can be seen from the wide range of services delivered. Two VHH case studies are presented in Figure 3 to provide a summary of the nature and frequency of the treatment given to the clients. Both clients had more than one GP session, and one client booked both GP and psychologist sessions. Health problems included self-care issues such as poor sleep, physical concerns such as chronic lower back pain, migraine, broken tooth, skin lesions, pulmonary disease, psychological conditions associated with being a survivor of domestic violence and having a depressed mood disorder, and lifestyle problems such as smoking. The services provided included medical prescriptions, psychological therapy such as emotional regulation and problem-solving strategies, referral to dentist and specialists, assistance to fill out social housing medical and disability parking forms, and healthy lifestyle education such as advice on sleep hygiene practice and smoking cessation.

#### 3.1.2. Acceptability and Appropriateness

According to the service provider interviews, the VHH has been well-accepted by the clients and healthcare providers involved. One service provider described the model as being ‘comfort in their own homes’ for the clients as they do not have to seek a GP outside the crisis accommodation setting. The technological infrastructure and the assistance from the project nurse and case managers were identified as making the model appropriate for people experiencing homelessness to ensure they can use it conveniently.

*When you’re homeless, you don’t want to see a doctor, everybody hates you. This way [people experiencing homelessness] can see that the doctors and everybody else wants to help them. … it’s great that the psychologists have come on board because homeless people have got problems, worries and things like that.*

(Lived experience client)

*A lot of our people have some sort of cognitive disability … so need a bit of support to [attend the clinic] … So having the caseworkers and that nurse on site preparing the person to chat to the doctor is really great. The privacy as well, you know that it’s a safe space for that client to talk to a doctor. And obviously there’s consent about-sometimes a caseworker will go in but very rarely. They’ve built really good trust with the doctors.*

(Management)

#### 3.1.3. Sustainability

The sustainability of the model is the extent to which it is maintained within the service setting. The management at the crisis accommodation setting noted that the VHH model had become part of their routine care. For instance, when case managers noticed someone was feeling unwell, they started to suggest scheduling a VHH appointment.

*People have started to get to know that there’s a doctor that they can see, and so they will ask quite often to see the doctor. If we notice someone’s a bit unwell, we would say, ‘How about we book you into see the virtual hospital?’*

(Management)

The management had identified that there was poor access to GPs and specialists in the local area, so the VHH enabled convenient access to health care.

*Bulk billing, there’s not a lot of bulk bill doctors. So the cost of seeing specialists; our clients couldn’t afford that. So cost effectiveness for the client, and I guess for us it’s around that we are a small organisation, we can’t afford to pay for a doctor to be on site and that’s the most effective thing for our client, to have access to medical support on site.*

(Management)

However, there were concerns related to the sustainability of the VHH program, particularly the certainty of having continued funds to employ the project nurse to assist the VHH.

*So let’s just say hypothetically, we get told this pilot’s ended and we can’t make it work without the nurse, then at least we’ve got that link to local area health district and link to a medical professional. We’d probably be looking at putting in grants for something more, so whether that is to fund a nurse to run rpavirtual here on site, or whether that is to look for a new GP.*

(Management)

#### 3.1.4. Fidelity to Model of Care

The fidelity of the model is the degree to which it was implemented according to the original plan. As stated, the VHH was designed to complement the regular on-site GP services. However, it evolved to become the only way for clients to access primary care in the crisis accommodation setting. The new service was considered pragmatic in response to the changing client needs, and there was agreement that it was performing very well and achieving the intended outcomes.

*The model of care and service that we’re actually delivering now is very, very different from what was originally proposed … What I’m informed is: the client experience of receiving care and the relationship with the collaborator, the Foundation; I get very positive feedback. Everyone is speaking very highly of the service and the work that it’s doing and the importance of the work that it’s doing, even though it’s quite different from what was originally conceived.*

(Management)

### 3.2. Service and Client Outcomes

The service and client outcomes of accessibility, technological infrastructure, quality of care, stakeholder experiences, impacts, and cost were evaluated using PREM data and stakeholder interviews.

#### 3.2.1. Accessibility

PREMs and qualitative data indicated that there was improved accessibility to health care, which was affordable, convenient, and acceptable for people experiencing homelessness in the crisis accommodation.

According to PREM data, if clients did not use the VHH, 50% reported that they would have tried to see another GP/health professional, 35% would not have sought treatment, 15% did not know, and nil reported that they would have gone to hospital. Outside the VHH, 55% reported that they have a current GP that they see regularly, and 40% do not have a regular GP. In addition, 45% had seen a GP within the last month, 20% had seen a GP 1–3 months ago, 15% 3–6 months ago, 5% 6–12 months ago, 5% longer than 12 months, and 10% could not remember. 

All service providers indicated that the VHH provided affordable and convenient primary care and psychological services for people experiencing homelessness in the crisis accommodation setting.

*You make it easy for them, also they’re not good with timekeeping so … we shuffle around the clinic. It’s like, “Oh, this person’s here now, can you bring them in?” So you have to have these flexible models that understand this very specific group, that are so in need. They really have very complex, challenging, reoccurring healthcare needs.*

(Healthcare provider)

The health care provided considered the physical and complex social needs patients were facing. A significant portion of the VHH consultations with health professionals at the crisis accommodation setting addressed clients’ specific needs, such as filling in Department of Housing and National Disability Insurance Scheme forms.

*Sometimes that’s a long appointment, sometimes an hour to complete this (housing) form with the patient and the caseworker because it needs to be correct. Otherwise, I then get emails needing it changed, and that just adds to the work. But I’ve got quite good now, we’re very familiar with these forms.*

(Healthcare provider)

Staff at the crisis accommodation setting reported an increase in clients utilising psychological services, who they considered would not typically seek access to the service. The virtual format made access to psychological services easier, likely contributing to greater client engagement.

*That’s been a game changer really. These are people that would never go to a psychologist. Having it in a room that they feel comfortable in, … and setup, … it will break down the barrier for them to feel like seeing a psychologist isn’t that scary.*

(Management)

#### 3.2.2. Quality of Care 

The quality of care was assessed through PREM and interview data, which revealed a high level of attainment at the VHH including in comparison to other virtual care services.

According to the PREM responses, 100% of clients rated the care provided by the VHH as excellent (70%) or good (30%) and that their privacy was maintained; 35% strongly agreed, 50% agreed, and 10% were undecided that the virtual appointments were the same or better than traditional in-person appointments; 90% felt they were always treated with respect and dignity by the virtual health clinicians, and 10% reported that they were mostly treated with respect; 84% felt their views and concerns were always listened to, 11% reported they were mostly listened to; 85% reported that they felt they were always included in making decisions about their condition and care needs as much as they wanted and 15% were mostly involved; 68% strongly agreed that virtual care clinicians explained things in a way they could understand, and 32% agreed.

PREMs comparison between VHH at Haymarket and other virtual care services

The PREMs results for clients who received health from the VHH were compared to the PREMs results of people who received care from other virtual care services provided at rpavirtual. The details of the comparison are included in Table 4. In general, compared to people using virtual care services at rpavirtual, people experiencing homelessness using VHH experienced similar perceptions of care as those in other rpavirtual virtual care services. These measures included the quality of the care, that the service they received helped them and met their needs, they felt involved in decision making, they were treated with dignity, their concerns were being listened to, and that they perceived that virtual care appointments were the same or better than a traditional in-person appointment. Further statistical analysis using Chi-square methods indicated there was a significant difference in the agreement that privacy was maintained when accessing virtual health care between health professionals and people experiencing homelessness (100%) compared to the general population (69%) (*p* ≤ 0.001).

In the interviews with case managers and clinicians, they agreed that the principle of person-centred and holistic care can be well-maintained within a virtual delivered format. They also noted that this approach has led to rewarding client-clinician experiences.

*We really give these patients time, and we’re aware that they have complex needs … and often that’s what people need … I’m seeing them week after week, following them up. They like it, we develop rapport, they’re given time. We know they’re complex, we don’t rush them. We work with them.*

(Healthcare provider)

Additionally, management noted that there was higher than expected demand for and attendance at psychologist appointments.

*We initially limited the psychology service to four sessions per week, but we now have more clients than we thought. … some clients are only able to be seen every third week rather than every second week. … [and] that does compromise therapeutical best practice. … there’ll be less intervention …*

(Management)

Clinicians reported that although the VHH format allowed them to assess most client needs effectively, the inability to perform some physical examinations may mean some referrals to other services.

*We can’t physically put our hands on them in the sense of certain examinations … The limitations of virtual are, you probably increase your referral to other services because you can’t see things because you’re not there in person.*

(Healthcare provider)

Case managers explained that while they could encourage clients to adhere to their medical regimes and monitor their action, they could not enforce compliance with taking medicine as prescribed.

*What we do is we put it in front of them with a glass of water and we monitor them taking it. And then we record what they’ve taken and if they take too many, we just write, “Took one extra against staff advice” because we get to know what our clients take and if they go to take two or more than what they should. … then we just note it in the case notes, and we note it in the location chart that they’ve taken extra (medication) against advice.*

(Case manager)

#### 3.2.3. Outcomes and Impact 

According to PREMs, 100% of the clients felt their VHH appointment had benefited them. Among 20 clients, 55% strongly agreed, and 40% agreed that the care and treatment received helped them.

Clinicians and management further perceived that the impact of the VHH reduced unnecessary hospital presentations and the number of people forgoing healthcare needs.

*… to have a service that is at their residence that the Haymarket staff help facilitate and organise and book the appointments, means that their ability to attend is going to be higher. … a few of them do still see other GPs, but a lot of them either don’t—so just perhaps ignore their health or wait until things get dire, … go to ED is generally what happens…*

(Healthcare provider)

Case managers and clinicians reported some concern about the ability of clients to access GP services and psychological treatment after being discharged from short-term accommodation.

*Because at the moment the clinic is only available to residents, if they were to move out into alternate accommodation, they’re not able to continue those psychology sessions, at the moment. Hopefully that will be explored because it would be amazing if there’s a bit more interim support once they move out.*

(Case manager)

#### 3.2.4. Technological Infrastructure 

Clinicians and case managers described the technical infrastructure as being convenient to use. However, a few technical difficulties occurred early in the virtual care setup, delaying the GP and psychologist consultation sessions. According to PREMs data, three clients (15%) experienced technical issues during their VHH appointment, including poor sound and IT connection issues. The clinicians found the role of the project nurse important to ensure IT connectivity.

*The IT connection isn’t great, but that’s probably a minor thing. It’s very helpful having a Nurse Unit Manager [NUM] facilitate the clinic because some of these patients may not be familiar with using an iPad, so it can only really run virtually with a NUM or a nurse on site.*

(Healthcare provider)

#### 3.2.5. Stakeholder Experiences

Stakeholder experiences of the implementation of the VHH include the level of engagement and collaboration among partners and the target population to identify opportunities for improvement. All participants indicated that there had been an active partnership and participation from the staff and partners of the Homeless team, crisis accommodation, and rpavirtual. An active partnership started in the development stage and continued into the implementation stage.

*We’ve had good engagement from [the crisis accommodation setting] from the beginning and even at the preliminary stage where were preparing the pitch. So we all did it together as one group.*

(Management)

*…being able to liaise with and collaborate with (the project nurse), the doctors and the psychologist has been really, really open and an easy collaboration. I can’t think of anything that could be improved in that way.*

(Case manager)

The GP and psychologists reported that the VHH model of care offered service delivery flexibility and the potential for expanding the service provided.

*The initial plan was not to necessarily have psychology; it was to have the nurse and GP in there, which would help the health system out. But then …they got some virtual psychologists,…. So here we came on board and supported them.*

(Healthcare provider)

Some service providers in management also discussed that the development of the VHH model of care was complex. There needed to be adequate time for discussion, clear communication channels, and understanding of respective roles and responsibilities.

*…we know we need to do that very carefully with very clear documentation about escalation pathways, roles and responsibilities, clinical governance eligibility criteria, all that stuff. … We’ve needed to take the time to get those things right. But we got there, we got the model of care confirmed.*

(Management)

#### 3.2.6. Examination of Costs

The cost of the VHH model was evaluated in relation to resource utilisation, appointment management expenditure, and other factors. The crisis accommodation reported that the VHH service has improved its model of access to primary health care. Without the VHH service, they would have to recruit a GP (which to date has been unsuccessful), allocate funds for an on-site GP and medical supplies, as well as provide extra costs to assist clients in accessing GPs outside the crisis accommodation setting. Moreover, the VHH has reduced clients’ out-of-pocket health spending to primary care.

*It’s been cost and resource effective because there’s not a lot of bulk bill doctors … the cost of seeing specialists, our clients couldn’t afford that. … we are a small organisation. We can’t afford to pay for a doctor to be on site and that’s the most effective thing for our clients; to have access to medical support on site. For us to support someone that needs extra support and take them out to a doctor, we can’t always do that because we have a rostering system … for us cost-wise, (the VHH)… is really helping. I could work it out for you, but we would be saving hundreds of dollars each client.*

(Management)

From the healthcare providers’ management perspective, the utilisation and cost for the psychological service exceeded the initial estimate. Healthcare providers also mentioned that they spent longer contacting and tracking the clients’ medical histories from other hospitals and assisting clients with housing form applications.

*They’re very complex patients. … the team have estimated that each patient requires two to three times the amount of indirect care of other rpavirtual patient cohorts. But we would expect that from this group. (The psychology service) equates to just over one 8-h working day for two clients. We initially limited the psychology service to four sessions per week, but we now have more clients than we thought.*

(Management)

*I usually have an initial appointment with them because I have to gather information … sometimes they don’t have any records, I have to collect records from other hospitals, which again takes a lot of time, contacting the other hospitals, trying to get the records … second appointment, usually with the caseworker to complete the housing forms … sometimes that’s a long appointment, sometimes an hour to complete this form with the patient and the caseworker because it needs to be correct.*

(Healthcare provider)

Management identified a potential resource improvement by shifting the role of project nurse to focus on clinical work while the caseworker could assist with the VHH equipment setup.

*From a cost effectiveness perspective, it’s a very expensive use of her (the project nurse’s) time. I think she’s paid at a NUM level. I would like to see [the crisis accommodation setting] be able to assist with that (to connect with a practitioner online), with their on-site staff. That would make … a much more cost-effective model and we could use the nurse to do more useful clinical work.*

(Management)

A further examination of operational and intervention costs was highly recommended.

## 4. Discussion

### 4.1. VHH Implementation Success

The evaluation of the VHH for people experiencing homelessness in crisis accommodation demonstrated its feasibility. This was demonstrated through the interrelationships between implementation and service and client outcomes [29]. Despite pragmatic deviations from the original program design, the new VHH model has shown a positive implementation impact regarding its adoption, acceptability, client satisfaction, appropriateness, and sustainability.

When evaluating implementation outcomes in a pilot project, it is beneficial to assess whether the program is applicable to different populations and contexts. It is important not to assume that all effective interventions are suitable for every context and setting [42]. Implementation frameworks focus on the elements of acceptability, appropriateness, fidelity, adoption, and sustainability of the program to provide insights into the contextual feasibility and success of the program [29]. This study indicated that people experiencing homelessness were willing and comfortable seeing a GP and psychologist virtually and receiving the same benefits as the general population when supported by a project nurse. The VHH service is likely to be easily replicated for other people experiencing homelessness outside the crisis accommodation setting, thereby extending the benefits of the VHH.

### 4.2. Impact on People Experiencing Homelessness

This study’s use of qualitative and quantitative data highlighted the alignment between the stakeholder and client experiences regarding acceptability and improved access to health care through the VHH. The PREMs data indicated that recipients perceived that the treatment was of high quality, it was carried out respectfully, they felt involved in decision making, and that it was beneficial and private. Interestingly, if clients did not use the VHH, 50% reported that they would have tried to see another GP/health professional, and 35% would have not. The interviews highlighted the model’s acceptability and accessibility. Case study vignettes demonstrated that a significant part of VHH consultations with health professionals addressed specific client needs, such as filling in the Department of Housing and National Disability Insurance Scheme forms. This indicates the service has not only improved access to primary health care but also impacted on the accessibility of other services such as NDIS, housing support, and specialist secondary health care.

As indicated in the case studies, PREMs, and stakeholder interviews, the flexibility of this outreach service and its ability to meet clients’ basic needs and referrals were key features in removing barriers to accessing primary health care for this population group, as demonstrated in other studies [43]. The healthcare providers in this study delivered medical assessments, treatments, and prescriptions; psychological assessments and treatments; referral services to dentists and specialists; lifestyle modification education such as smoking cessation, and assistance in completing social housing and disability forms. These services were provided free of charge for clients through rpavirtual.

According to PREM data, there is an interesting finding in this study about clients’ perceptions of their privacy. People experiencing homelessness have been reported to experience a lack of privacy due to the inexistence of their own personal space [44] and the need to have privacy and confidentiality when using virtual technology [26]. The lack of privacy may also occur when accessing healthcare due to this situation. Unexpectedly, clients accessing the VHH within the crisis accommodation reported that they felt that VHH clinicians maintained their privacy, which was significantly higher compared to the general populations’ perception of privacy in accessing virtual care. The findings in this article are important for homelessness health services, as virtual health care delivery can create a sense of privacy when a person’s personal space may have been compromised due to their living arrangements.

The service providers’ interviews highlighted the high utilisation of psychology sessions in the VHH service. Before the introduction of VHH, the crisis accommodation staff indicated that very few people experiencing homelessness saw a psychologist for their mental health issues because of a range of factors, including poor access and the lack of perceived benefits of psychological counselling. This situation is in contrast to the research indicating people seeking specialist homelessness services have a high prevalence of mental health conditions [5]. Since the VHH implementation, the service reported that many clients were given their first experience of a psychological session, and this accessibility has increased requests for more psychological sessions.

Bennet-Daly et al. [45] reported that people experiencing homelessness often do not prioritise their healthcare needs because they tend to focus on fulfilling their basic daily needs such as food, security, and shelter, often due to their financial constraints. This situation may make them unable to commit to booking and attending GP appointments. Meanwhile, VHH stakeholders’ interviews revealed that the VHH may have prevented clients from forgoing healthcare needs and reduced unnecessary hospital presentations. This reinforces other research indicating that, given the opportunity to receive necessary health care, clients would want to manage their chronic health conditions better [6]. In this sense, the financial constraint to accessing healthcare services has been removed because VHH is provided free for those residing in crisis accommodation.

### 4.3. Use of Digital Health in Homeless Populations

This study contributes to existing knowledge of the utilisation of digital primary health care for people experiencing homelessness by adopting an implementation outcome framework. The framework assists in understanding to what extent the digital health intervention that facilitates accessibility is acceptable, appropriate, and feasible, and how it could be sustained in crisis accommodation. It is important that public health practitioners do not assume that these aspects of health program implementation would be similar between the general population and vulnerable population. Moreover, this study also unravels the high demands of seeing psychologists among people experiencing homelessness with a mental health condition. This high demand can be considered an unanticipated need. The VHH filled these needs of addressing mental health issues and benefited the first experience of psychological service for this specific population.

### 4.4. Barriers and Facilitators to Implementation

The implementation success of VHH in the crisis accommodation setting had some driving facilitators. Firstly, the strong partnership between SLHD’s Homeless program and rpavirtual in SLHD and the crisis accommodation service since the development of the model showed the adaptive and flexible collaboration of partners in response to changing situations. Secondly, the common mission of all service providers, including the GP and psychologists, to provide accessible, equitable primary care for people experiencing homelessness was well-aligned. Additionally, most GPs and psychologists who were involved in the VHH have a passion for working with people experiencing homelessness, which contributed to the cultural sensitivity and appropriateness of the service [46,47]. This aligns with previous research on intersectoral responses to homelessness [48].

Another facilitator was the implementation lead role held by the project nurse, who was instrumental in delivering the VHH service, collaborating with case managers in the crisis accommodation setting, and providing health care by rpavirtual. Other studies have highlighted the importance of on-site assistance to ensure the efficiency of delivering virtual health for people experiencing homelessness [22]. Lastly, having no out-of-pocket expenses for the GP and psychologist service provided by rpavirtual has reduced the financial barriers to the client so they can effectively access the service.

Regarding barriers to implementation, the temporary nature of client stays (in crisis accommodation) means there is a risk of discontinuity of care, particularly for follow-up care, once clients leave the accommodation setting. Notably, case conferencing occurred, which helped in planning for continuity of care post-exit from the crisis accommodation setting. In relation to IT infrastructure, the technological aspects of the service did improve and became a non-significant barrier. Other limitations include a full examination of program costs that were beyond the scope of the project and the absence of a clinical outcome evaluation.

## 5. Limitations

The project was a pilot evaluation, so there are some limitations to the scope. The manuscript lacks detailed accounts of people with lived experience, data on long-term or pre/post-client health outcomes, and whether there was any continuity of physical and psychological care. Future studies could examine long-term outcomes and compare them with a matched data/control group.

## 6. Conclusions

The evaluation of the VHH for people experiencing homelessness in crisis accommodation settings has demonstrated its feasibility and positive impact on clients. Clients reported benefiting from the VHH service, and service providers believed that the VHH also reduced unnecessary hospital presentations and prevented clients from forgoing their healthcare needs. The service achieved acceptability and appropriateness despite some adjustments to the model of care. The VHH has improved access to GP and psychology services for people experiencing homelessness as well as enhancing access to social care services such as NDIS and housing support. The findings indicate that the service can maintain person-centred care and the privacy of clients in the virtual delivery of healthcare supported by an on-site implementation team.

Key implementation facilitators include strong collaboration and engagement among service providers and the project nurse. The temporary nature of clients’ stays in crisis accommodation posed a barrier to continuity of care. However, strategies like case conferencing helped mitigate these risks.

There has been limited research conducted on the use of virtual primary health care with populations experiencing homelessness with chronic conditions. This evaluation presents innovative research conducted with a vulnerable population with complex health needs. The small number of clients in the quantitative studies over a relatively short period of time provides important findings to assess the project, but caution should be applied to generalising to all similar population groups. Some limitations of the study include that a clinical outcome evaluation was not conducted and that a full examination of cost was beyond the scope of the project. There were some limitations with the extensiveness of the assessment and program changes.

Overall, the VHH model has provided accessible and culturally sensitive healthcare to individuals experiencing homelessness. Future research should focus on comprehensive cost evaluations and clinical outcomes to further validate the model’s efficacy and sustainability.

## Figures and Tables

**Table 1:** The use of data sources to assess the implementation, service, and client outcomes.

Methods	Data Source	Respondents	Implementation Outcomes- Related Data Source	Service–Client Outcomes- Related Data Source
Quantitative analysis	VHH appointment records	33 clients accessing VHH	Adoption, sustainability	Cost efficiency
Qualitative analysis	PREMs	20 clients completed PREMs	Fidelity	Accessibility, IT infrastructure, quality of care, impact
	Stakeholders’ interviews	12 interviews, including management, case managers, healthcare providers, and lived experience of homelessness	Acceptability, appropriateness, sustainability, fidelity	Accessibility, IT infrastructure, quality of care, impact, stakeholders’ satisfaction, cost efficiency

**Table 2:** The role of the implementation team who interacted with clients.

Implementation Team	Role
Project nurse	Manage appointments, setup VHH equipment, and measure vital signs of clients on the site. This nurse was on site during appointments for the initial stage of the project.
Case managers	Suggest clients attend VHH when health concerns occur, provide emotional support, and remind clients to take medication.
GPs	Provide general practice consultation, prescription, and client referral.
Psychologists	Provide psychological consultation, action plan, and client referral.

**Table 3:** Characteristics of PREMs respondents (*N* = 20).

Characteristics	*N*	%
Gender		
Woman	11	55
Man	9	45
Non-binary	0	0
Prefer not to say	0	0
Age group		
18 to 34	<5	15
35 to 44	10	50
45 to 74	7	35
75 or older	0	0
Language spoken at home		
English	17	89.5
Language other than English	2	10.6

**Table 4:** PREM comparison between VHH at the Haymarket and other virtual hubs.

Question	Virtual Health Hub			Other Virtual Care Centres			*p*-Value
	Strongly Agree	Agree	Combined	Strongly Agree	Agree	Combined	
Overall, care received rated as good or very good	70.0% (14)	30.0% (6)	100%	82.1% (216)	16.3% (43)	98.4%	0.27
The care and treatment received helped them	55.0% (11)	40.0% (8)	95%	63.5% (155)	32.8% (80)	96.3%	0.75
rpavirtual met their needs	60.0% (12)	35.0% (7)	95%	79.6% (195)	18.0% (44)	97.6%	0.13
They felt they were involved as much as they wanted in making decisions about care and treatment	85.0% (17)	15.0% (3)	100%	67.7% (178)	16.7% (44)	84.4%	0.14
The clinicians explained things in a way they could understand	68.4% (13)	31.6% (6)	100%	67.2% (162)	30.7% (74)	97.9%	0.82
They were treated with respect and dignity	90.0% (18)	10.0% (2)	100%	94.3% (231)	5.3% (13)	99.6%	0.66
Their views and concerns were listened to	84.2% (16)	10.5% (2)	95%	78.1% (193)	12.1% (30)	90.2%	0.78
My virtual care appointment was the same or better than a traditional in-person appointment	35.0% (7)	50.0% (10)	85%	36.9% (89)	39.4% (95)	76.3%	0.57
Their privacy was maintained	70.0% (14)	30.0% (6)	100%	63.9% (145)	4.8 (11)	68.7%	<0.001

## Data Availability

The data presented in this study are available on request from the corresponding author due to privacy reasons.
